# Microbes Associated with Freshly Prepared Juices of Citrus and Carrots

**DOI:** 10.1155/2014/408085

**Published:** 2014-10-16

**Authors:** Kamal Rai Aneja, Romika Dhiman, Neeraj Kumar Aggarwal, Vikas Kumar, Manpreeet Kaur

**Affiliations:** ^1^Vaidyanath Research, Training and Diagnostic Centre, Kurukshetra 136118, India; ^2^Department of Microbiology, Kurukshetra University, Kurukshetra 136119, India

## Abstract

Fruit juices are popular drinks as they contain antioxidants, vitamins, and minerals that are essential for human being and play important role in the prevention of heart diseases, cancer, and diabetes. They contain essential nutrients which support the growth of acid tolerant bacteria, yeasts, and moulds. In the present study, we have conducted a microbiological examination of freshly prepared juices (sweet lime, orange, and carrot) by serial dilution agar plate technique. A total of 30 juice samples were examined for their microbiological quality. Twenty-five microbial species including 9 bacterial isolates, 5 yeast isolates, and 11 mould isolates were isolated from juices. Yeasts and moulds were the main cause of spoilage of juices. *Aspergillus flavus* and *Rhodotorula mucilaginosa* were observed in the maximum number of juice samples. Among bacteria *Bacillus cereus* and *Serratia* were dominant. *Escherichia coli* and *Staphylococcus aureus* were detected in few samples. *Candida* sp., *Curvularia*, *Colletotrichum*, and *Acetobacter* were observed only in citrus juice samples. *Alternaria*, *Aspergillus terreus*, *A. niger*, *Cladosporium*, and *Fusarium* were also observed in tested juice samples. Some of the microorganisms detected in these juice samples can cause disease in human beings, so there is need for some guidelines that can improve the quality of fruit juices.

## 1. Introduction

Unpasteurized fruit juice is defined as the product produced by pressing or squeezing of the fruits [1]. Consumption of fresh juices increased dramatically due to their freshness, high vitamin content, and low caloric consumption [2]. Extracted juices from fruits contain most substances which are found in the original ripe and sound fruit from which the juice is made. The high potassium and low sodium characteristic of most juices help in maintaining a healthy blood pressure. Vitamin C is naturally present in juices which are essential for the body to form collagen, cartilage, muscle, and blood vessels. It also helps in the absorption of iron [3].

Fruit juices contain a microflora which is normally present on the surface of fruits during harvest and postharvest processing which include transport, storage, and processing [4]. Many microorganisms such as acid tolerant bacteria and fungi (moulds, yeasts) use them as a substrate for their growth. Yeasts form the main flora of fruits before processing because of acidic pH. The major genera include* Candida*,* Dekkera*,* Hanseniaspora*,* Pichia*,* Saccharomyces*, and* Zygosaccharomyces*.* Penicillium*,* Byssochlamys*,* Aspergillus*,* Paecilomyces*,* Mucor*,* Cladosporium*,* Fusarium*,* Botrytis*,* Talaromyces*, and* Neosartorya* are filamentous fungi most frequently isolated from fresh fruits and juices. Among bacteria, lactic acid bacteria and acetic acid bacteria have been isolated from fruit juices [5].

The critical factors affecting the spoilage of juices include juice pH, oxidation reduction potential, water activity, availability of nutrients, presence of antimicrobial compounds, and competing microflora. Among these factors, pH and water activity are the most influential factors affecting the spoilage of juices. The spoilage caused by microorganisms in juices includes cloud loss, development of off-flavours, CO_2_ production, and changes in colour, texture, and appearance resulting in degradation of product [6, 7]. The most commonly reported bacterial genera include* Acetobacter*,* Alicyclobacillus*,* Bacillus*,* Gluconobacter*,* Lactobacillus*,* Leuconostoc*,* Zymomonas*, and* Zymobacter*. Among yeasts* Pichia*,* Candida*,* Saccharomyces*, and* Rhodotorula* are commonly encountered genera responsible for spoilage of juices [8]. Certain common moulds such as* Penicillium* sp.,* Aspergillus* sp.,* Eurotium*,* Alternaria*,* Cladosporium*,* Paecilomyces*, and* Botrytis* have also been reported in spoilage of fruit juices [5, 6].

Fruit juices have pH in the acidic range (<4.5) serving as important barrier for microbial growth. However, food borne pathogens such as* E. coli* and* Salmonella* survive in acidic environment of fruit juices due to acid stress response. Therefore, in the last two decades a number of food borne outbreaks associated with unpasteurized fruit juices have been documented in many countries [1, 9]. The source of entry of microorganisms into fresh fruit juices from environment exposure and soil. In developing country like India, a large population of all income and age groups consume freshly squeezed fruit and vegetable juice [10], but the presence of pathogenic microorganisms in street vended fruit juices has also been reported in various parts of India such as Vishakhapatnam [11], Mumbai [12], Amravati [13], and Nagpur [10]. In view of the demand for fresh fruit juices throughout the year and threat of emerging food borne outbreaks associated with consumption of fruit juices, the aim of present study was to investigate the microbiological examination of freshly prepared juices commonly consumed in Kurukshetra.

## 2. Materials and Methods

### 2.1. Fruit Juice Preparation

Three juices commonly consumed in Kurukshetra such as orange (*Citrus reticulata* Blanco), sweet orange (*Citrus sinensis*), and carrot (*Daucus carota*) were selected for microbiological study. Sweet orange, carrot, and orange were purchased from the local markets of Kurukshetra from October 2011 to February 2012. Each sample was washed, peeled, and cut into pieces and juice was extracted through sterile hand blender and poured into sterile beaker.

### 2.2. Measurement of pH

The pH of juice samples was measured using a pH meter.

### 2.3. Microbiological Analysis

The microbiological study of fruit juices was done by serial dilution agar plate technique. Ten mL of juice sample was diluted with 90 mL of 0.1% sterile peptone water (1 g peptone, 1L distilled water) and plated on nutrient agar (pH 5.5) for enumeration of bacteria and PDA supplemented with antibiotic (pH 5.5) for enumeration of fungi in duplicates [4]. Uninoculated plates of PDA and NA were used as control. Mould and yeast isolates were purified on potato dextrose agar, bacteria on nutrient agar, and further subcultured for microscopic examination and identification.

### 2.4. Identification of Bacteria

For bacterial identification, 24-hour-old culture of bacteria was observed under microscope by gram stain method and further various biochemical tests were performed for the identification of bacteria such as catalase test, oxidase test, starch hydrolysis test, sugar fermentation test, IMViC test, and methods described in “Bergey's Manual of Systematic Bacteriology” [14]. Further identification of bacteria was performed on the basis of methods described in “Compendium of methods for the microbiological examination of foods” [15, 16].

### 2.5. Identification of Yeasts

The methods adopted for identification of yeasts include morphological characteristics, fermentation of sugars, germ tube test and cycloheximide resistance test, and methods described in “Fungi and Food Spoilage” [17, 18].

### 2.6. Identification of Moulds

Moulds were identified on the basis of morphological and cultural characteristics such as colour of the colony, surface, appearance, presence, and absence of cross walls, and asexual and sexual reproductive structures. Further identification of moulds was carried out according to the methods described in “Fungi and Food Spoilage.” Moulds were cultured on Czapek yeast extract agar (pH 6.7), Malt extract agar (pH 5.6), and Glycerol nitrate agar (pH 7.0) at 25°C.

## 3. Results and Discussion

In the present study, 30 samples of freshly prepared juices (10 samples each of orange, sweet orange, and carrot) were examined for microbiological analysis. The pH range of juices is shown in Table 1. Factors which determine the colonization of juices by microorganisms include pH, redox potential, water activity, nutrients, structures, antimicrobial agents, temperature, relative humidity, and atmosphere [1]. In the present study the frequencies of occurrence of moulds and yeasts were more as compared to bacterial genera which is attributed to low pH values and high sugar content [19].

A total of 34 bacterial, 12 yeast, and 25 mould isolates were isolated from juices classified by grouping them into 9 bacterial species, 5 yeast species, and 11 mould species on the basis of phenotypic characteristics. Morphological and biochemical properties of bacteria were explained in Tables 2 and 3. Details of morphology and physiology of yeasts were described in Tables 4 and 5. Colonial and microscopic characteristics of various moulds were summarized in Tables 6 and 7.

Yeasts and moulds are capable of growth at pH values of 1.5 and at water activity values below 0.89. The minimum pH values allowing the growth of lactic acid bacteria (pH 2.9–3.5), acetic acid bacteria (pH 3.0–4.5), and enteric bacteria (pH 3.6–4.5) are higher than those for growth of yeasts and moulds [6].

The frequency of occurrence of bacteria, yeasts, and moulds are summarized in Tables 8, 9, and 10, respectively. The occurrence of bacterial genera ranged from 10% to 56% (Table 8).* Bacillus cereus* and* Serratia* sp. were detected in a greater number of samples.* Bacillus cereus* was also observed in 64.91% of samples of unpasteurized street vended fruit juices [20].* Leuconostoc* and* Lactobacillus* were also reported as important group of spoilage microorganisms in acidic products [21]. The presence of lactic acid bacteria more frequently occurs in unpasteurized juices [22]. These microorganisms produce acetic and formic acids along with ethanol and carbon dioxide which can alter the flavor of juice [23].* Leuconostoc*,* Lactobacillus*, and* Acetobacter* were detected in tested juice samples (Table 8).

The presence of* E. coli*,* Salmonella*, and* S. aureus* in fruit juices is primarily concern because these pathogens were implicated in a number of outbreaks associated with fruit juices [1]. In our study, the presence of* E. coli* and* S. aureus* was detected in a smaller number of samples. The survival of pathogens in acidic environment of juices is attributed to their ability to regulate their internal pH and maintained at neutral pH by combination of passive and active homeostasis mechanisms [24]. The acid survival mechanisms of enteric bacteria are due to induction of enzymes that are involved in raising the internal pH and activation of enzymes devoted to the protection and repair of proteins and DNA [25].

Yeasts genera responsible for spoilage of fruit juices include* Candida*,* Pichia*,* Rhodotorula*,* Torulopsis*,* Saccharomyces*,* Zygosaccharomyces*,* Hansenula*, and* Trichosporon* [26]. In our study, the dominant yeasts isolated from juices were* Rhodotorula*,* Pichia*, and* Saccharomyces* (Table 9). Rhodotorula was found in maximum number of juice samples tested followed by* Pichia* and* Saccharomyces*.* Candida parapsilosis* and* C. krusei* were only detected in orange and sweet orange juices, not detected in carrot juice. Ghenghesh et al. [9] also reported the presence of* Candida* sp. in 58% of orange juice samples.* Rhodotorula*,* Pichia*,* Candida*, and* Saccharomyces* have also been reported as spoilage causing organisms in pasteurized fruit juices [4, 27]. Yeast spoilage in fruit juices is characterized by formation of CO_2_ and alcohol. Yeasts may also produce turbidity, flocculation, pellicles, and clumping. Yeasts also produced pectin esterases which degrade pectin causing spoilage; organic acids and acetaldehyde, which contribute to a “fermented flavor,” may also be formed [5, 6].

The dominant moulds recorded in fruit juices belong to* Penicillium* sp.,* Cladosporium* sp.,* Aspergillus niger*,* A*.* fumigatus*,* Botrytis* sp., and* Aureobasidium pullulans*. They produce mycelial mats and musty, stale off-flavours in juices [6].* Rhizopus* and* Mucor* are also associated with spoilage of fresh fruits and vegetables [28]. In the present study, the most frequently encountered moulds were* Aspergillus flavus*,* A. terreus*, and* Penicillium islandicum* (Table 5).* P. digitatum*,* Colletotrichum*, and* Curvularia* were isolated from orange and sweet orange juices.* Geotrichum* was detected in orange and carrot juice. Spoilage by moulds in fruit juices is characterized by loss of juice cloud [6]. Among these, some moulds produce mycotoxins which are of great threat to human health. Major mycotoxins associated with fruit juices are byssochlamic acid (*Byssochlamys fulva*,* B. nivea*), patulin (*B. fulva*,* B. nivea*, and* P. expansum*), ochratoxin (*Aspergillus carbonarius*), and citrinin (*Penicillium expansum*,* P. citrinum*) [29, 30].

## 4. Conclusion

Juices squeezed from fresh fruits and vegetables contain microorganisms which are potentially hazardous to public health. Juices were spoiled with high level of moulds and yeasts which is attributable to low pH of juices. The presence of pathogenic microorganisms in juices is clearly indication of food borne outbreaks. The selling and consumption of juices are never stopped on nutritional grounds as well as livelihood of street vendors. It is alarming situation for suitable agency to take some necessary action, make guidelines to prevent potential food poisoning from juices that contain pathogenic bacteria, and find natural antimicrobials from plants that control spoilage and pathogenic microorganisms in juices.

## Figures and Tables

**Table 1:** pH values of juices.

Juices	pH range	Mean
Orange	4.19–4.50	4.34
Sweet lime	4.70–5.47	5.08
Carrot	5.76–6.03	5.89

**Table 2:** Morphological characteristics of bacterial isolates of juices.

Bacterial isolates	Colour on nutrient agar	Configuration	Margin	Elevation	Gram reaction	Shape of isolate	Endospore staining
*Bacillus subtilis *	White	Circular lobate	Irregular	Flat	Positive	Rods in chains	Central spore
*B. cereus *	Off-white	Circular	Entire	Convex	Positive	Rods in chains	Central spore
*Escherichia coli *	Mucoid	Circular	Entire	Slightly raised	Negative	Rods	—
*Serratia *	Mucoid	Circular	Entire	Umbonate	Negative	Rods	—
*Leuconostoc *	Light yellow	Circular	Entire	Convex	Positive	Cocci	—
*Micrococcus *	Bright Yellow	Circular	Entire	Convex	Positive	Cocci shape in tetrad	—
*Staphylococcus aureus *	Golden yellow colour	Circular pin head colonies	Entire	Convex	Positive	Cocci in grapes like bunches	—
*Lactobacillus *	White	Circular	Entire	Raised	Positive	Rods	—
*Acetobacter *	Pale	Circular	Entire	Flat	Negative	Rods	—

—: absent.

**Table 3:** Biochemical characteristics of bacterial isolates of juices.

Bacterial isolates	Catalase	Oxidase	Starch hydrolysis	IMViC test				Sugar fermentation			
				Indole	Methyl red	Voges-Proskauer	Citrate	Glucose	Lactose	Mannitol	Sucrose
*Bacillus subtilis *	+	−	+	−	−	+	+	A	−	A	A
*B. cereus *	+	−	+	−	−	+	+	A	−	A	−
*Escherichia coli *	+	−	−	+	+	−	−	A + G	A + G	−	−
*Serratia *	+	−	−	−	−	−	+	A	−	+	−
*Leuconostoc *	−	−	−	−	−	+	+	A	−	A	−
*Micrococcus *	+	+	−	+	+	−	−	A	A	−	−
*Staphylococcus aureus *	+	−	−	+	+	−	−	A	A	A	A
*Lactobacillus *	−	−	−	−	−	−	−	A + G	A	−	−
*Acetobacter *	−	−	−	−	−	+	+	A	−	−	−

+: positive; −: negative; A: acid; A + G: acid + gas.

**Table 4:** Morphological details of yeast isolates of juices.

Yeast isolates	Colour on PDA	Configuration	Margin	Microscopic features
*Pichia *	Off-white	Hemispherical	Irregular	Ellipsoidal to cylindrical; reproducing by irregular budding
*Saccharomyces *	Off-white	Circular	Irregular	Spherical to subspheroidal; reproducing by irregular budding
*Candida krusei *	White	Circular	Irregular	Ellipsoidal to long cylindrical; reproducing by irregular budding
*Rhodotorula *	Pink	Circular or spreading	Regular	Ellipsoidal shape; reproducing by irregular budding
*Candida parapsilosis *	White to cream	Circular	Regular	Globose to ovoid budding

**Table 5:** Physiological tests for yeasts isolates of juices.

Yeast isolate	Germ tube test	Cycloheximide resistance	Sugar fermentation∗			
			Glucose	Sucrose	Lactose	Maltose
*Pichia *	−	+	+	+	−	−
*Saccharomyces *	−	−	+	+	−	−
*Candida krusei *	−	−	+	−	−	−
*Rhodotorula *	−	−	−	−	−	−
*Candida parapsilosis *	−	−	+	−	−	−

+: positive; −: negative; ∗fermentation means production of gas independent of pH changes.

**Table 6:** Morphological details of mould isolates of juices.

Mould isolate	Colony colour on PDA on front side	Colony colour on PDA on reverse side	Microscopic features
*Aspergillus flavus *	Yellow green	Colourless	Conidiophores arise separately from foot cell, phialides uniseriate and sometimes biseriate; conidia globose to subglobose

*A. terreus *	Brown	Colorless	Conidiophore borne from surface hyphae, stripes long, and smooth walled; vesicles with densely packed, short, narrow metulae and phialides; conidia unicellular, spherical, and very small

*A. niger *	Black	Creamy	Hyphae septate and hyaline, smooth walled conidiophores arising from foot cell; vesicles globose, whole vesicle fertile bearing two series of sterigmata; catenate conidia arranged in basipetal manner, unicellular, and globose

*Penicillium islandicum *	Ivy green	Creamy	Short conidiophores bearing a compact verticil of metulae, phialides closely packed in clusters bearing catenate conidia arranged in basipetal manner, conidia elliptical, smooth, and hyaline

*P. digitatum *	Green	Colourless	Conidiophores borne from surface and aerial hyphae with thin smooth walls; bearing terminal penicilli; terverticillate but frequently biverticillate or irregular

*Alternaria *	Black	Colourless	Small to large sized conidia with beak; arising in chains in acropetal manner with both transverse and longitudinal septa

*Cladosporium *	Black	Colourless	Conidiophore tall, dark upright, and branched variously near the apex, conidia 1-2-celled ovoid to cylindrical shape

*Colletotrichum *	Cottony white to pale gray mycelium	Colourless	Acervuli disc shaped, typically with dark spines or setae at the edge of conidiophores; conidiophores simple, elongate conidia single celled, hyaline or brightly coloured, cylindrical or pointed, straight or curved

*Curvularia *	Green to black	Black	Simple conidiophores bearing spores apically; Conidia dark, end cells, 3–5-celled; more or less fusiform, typically bent

*Fusarium *	Wooly white	Colourless	Conidiophores slender and simple, short or branched irregularly or bearing a whorl of phialides; conidia hyaline, variable, principally of two kinds, macroconidia several celled slightly curved or bent at the point ends, microconidia 1-celled, ovoid or oblong, borne singly or in chains

*Geotrichum *	White	Colourless	Conidia borne solely by the breakup of hyphae to form arthroconidia

**Table 7:** Colonial characteristics of different moulds' isolates of juices on CYA, MEA, and G25N media.

Mould isolate	Colony colour on CYA		Colony colour on MEA		Colony colour on G25N	
	Front side	Reverse side	Front side	Reverse side	Front side	Reverse side
*Aspergillus flavus *	Yellow green	Colourless	Yellow green	Colourless	Yellow green	Colourless

*A. terreus *	Brown	Dull brown	Brown	Dull brown	Brown	Dull brown

*A. niger *	Black	Pale to bright yellow	Black	Pale to bright yellow	Black	Pale to bright yellow

*Penicillium islandicum *	Greyish green	Orange to rust brown central area	Greyish green	Orange to rust brown central area	Greyish green	Orange to rust brown central area

*P. digitatum *	Greyish green to olive	Pale or brown	Dull yellow green	Pale or brown	Green olive	Pale

*Alternaria *	Grey to black	Black	Grey to black	Black	Grey to black	Black

*Cladosporium *	Olive to dark olive	Grey	Olive	Grey	Olive	Black

*Colletotrichum *	Grey	Pale grey	Grey	Pale grey	Black	Grey

*Curvularia *	Off-white to grey	Grey	Off-white to grey	Grey	Grey to black	Grey

*Fusarium *	White to grayish rose	Pale	White	Pale	White	Pale

*Geotrichum *	White	Pale	White	Pale	No growth	

**Table 8:** Percentage abundance of bacterial isolates in juice samples.

Bacterial isolates	Orange juice (*n* = 10)	Sweet orange juice (*n* = 10)	Carrot juice (*n* = 10)	Total number of samples	Percentage frequency
*Bacillus subtilis *	5	3	4	12	40%
*B. cereus *	8	4	5	17	56.7%
*Escherichia coli *	2	3	1	6	20.0%
*Serratia *	5	5	4	14	46.7%
*Leuconostoc *	4	5	—	9	30.0%
*Micrococcus *	—	3	—	3	10.0%
*Staphylococcus aureus *	2	1	2	5	16.7%
*Lactobacillus *	2	3	2	7	23.3%
*Acetobacter *	3	5	—	8	26.7%

—: absent.

**Table 9:** Percentage abundance of yeast isolates in juice samples.

Yeast isolates	Orange juice (*n* = 10)	Sweet orange juice (*n* = 10)	Carrot juice (*n* = 10)	Total number of samples	Percentage frequency
*Pichia *	8	7	4	19	63.3%
*Saccharomyces *	6	8	4	17	56.67%
*Candida krusei *	3	8	—	11	36.7%
*Rhodotorula *	9	6	9	24	80%
*Candida parapsilosis *	8	—	—	8	26.7%

—: absent.

**Table 10:** Percentage abundance of mould isolates in juice samples.

Mould isolates	Orange juice (*n* = 10)	Sweet lime juice (*n* = 10)	Carrot juice (*n* = 10)	Total number of samples	Percentage frequency = occurrence in observed samples/total no of samples
*Aspergillus flavus *	9	9	7	25	83.3%
*A. terreus *	5	7	4	16	53.3%
*A. niger *	3	2	1	6	20%
*Penicillium islandicum *	9	7	7	23	76.7%
*P. digitatum*	7	5	—	12	40%
*Alternaria *	6	5	3	14	46.7%
*Cladosporium *	3	4	2	9	30%
*Colletotrichum *	5	3	—	8	26.7%
*Curvularia *	3	2	—	5	16.7%
*Fusarium *	4	6	3	13	43.3%
*Geotrichum *	4	—	3	7	23.3%

—: absent.
